# HTLV-1-associated myelopathy/tropical spastic paraparesis (HAM/TSP)-like disease and toxic epidermal necrolysis (TEN)-like disease in HTLV-1 Tax transgenic mice

**DOI:** 10.1186/1742-4690-11-S1-P40

**Published:** 2014-01-07

**Authors:** Takeo Ohsugi, Makoto Wakamiya, Saki Morikawa, Kumi Matsuura, Toshio Kumasaka, Kazunari Yamaguchi

**Affiliations:** 1 Division of Microbiology and Genetics, Institute of Resource Development and Analysis, Kumamoto University, Kumamoto, Japan; 2 Department of Pathology, Japanese Red Cross Medical Center, Tokyo, Japan; 3 Department of Safety Research on Blood and Biologics, National Institute of Infectious Diseases, Tokyo, Japan

## 

Human T-cell leukemia virus type 1 (HTLV-1) can cause an aggressive malignancy known as adult T-cell leukemia/lymphoma (ATLL), as well as inflammatory diseases such as HTLV-1-associated myelopathy/tropical spastic paraparesis (HAM/TSP). We created a transgenic mouse model of HTLV-1 infection by using the distal promoter of Lck to express tax in mature thymocytes and peripheral T lymphocytes. Major phenotypes of disease in this model were mature T cell leukemia/lymphoma similar to ATLL and an inflammatory arthropathy. While expanding the transgenic mouse colony, we also found 2.7% of transgenic mice developed a HAM/TSP-like disease and 2.4% of transgenic mice developed a toxic epidermal necrolysis (TEN)-like disease. The HAM/TSP-like disease with symmetrical paraparesis of the hind limbs in transgenic mice was not inflammatory, unlike that found in HAM/TSP patients, but instead involved the invasion of histiocytic sarcoma cells into the lumbar spinal cord from the bone marrow where they had undergone extensive proliferation. Mice with the HAM/TSP-like disease showed abnormal expression of cytokines and chemokines, including TNF and IL-6. There are no reports of spontaneous symmetrical paraparesis caused by histiocytic sarcoma in mice. TEN is characterized by a rash, bullae, and diffuse exfoliation of wide cutaneous surface areas, as seen in second-degree burns. Massive keratinocyte apoptosis is the hallmark of TEN. The TEN-like disease in transgenic mice occurred mainly in C57BL/6 background strain mice. These mice showed a skin detachment rate of greater than 30%. The pathology in transgenic mice with a TEN-like disease is currently under study and will be discussed.

